# Verruca Vulgaris and Seborrheic Keratosis Exacerbated by Immunosuppression

**DOI:** 10.1155/2020/6682694

**Published:** 2020-12-02

**Authors:** Shohei Iida, Kyoko Sugioka, Makoto Kondo, Yoshiaki Matsushima, Kento Mizutani, Koji Habe, Keiichi Yamanaka

**Affiliations:** Department of Dermatology, Mie University, Graduate School of Medicine, 2-174 Edobashi, Tsu, Mie 514-8507, Japan

## Abstract

Verruca vulgaris is an infectious disease caused by the human papillomavirus and characterized by hyperkeratotic papules or plaques with a clear boundary. Seborrheic keratosis is a commonly encountered lesion on the face, trunk, or extremities and is described as seborrheic verruca because of its clinical similarity to warts; furthermore, it is occasionally associated with immune suppression, especially in cases of Leser-trélat syndrome. Although these diseases are frequently found in healthy individuals, they typically show a good response to cryotherapy. However, cases in immunosuppressed patients are intractable to therapy. Overall immune status is evaluated via complete blood count (CBC); however, white blood count does not show the exact immune ability, and NK cell activity is often decreased in cases of malignancy. Here, we present two cases of exacerbated verruca vulgaris and seborrheic verruca observed in patients with malignancy. Although the patients seemed to be in good condition and had a normal CBC, immunosuppression was suspected based on the degree of skin rashes. NK cell activity was decreased in both patients, and both cases had malignancy. The measurement of NK cell activity may be a useful approach to evaluate immune status.

## 1. Introduction

Verruca vulgaris is a benign proliferation of the stratified squamous epithelium resulting in a papillary or verrucous exophytic mass. Depending on the inoculation titer, an incubation period of 3 weeks to 8 months is required before lesions become apparent [1]. Lesions are easily seen on the hands, arms, and legs; however, they can also appear on the surface of the skin in any region of the body or, more rarely, on mucous membranes [2]. Seborrheic verruca is an asymptomatic benign epidermal keratinocytic tumor commonly seen in elderly patients. It may also act as an acquired cutaneous lesion predisposing individuals to the development of other dermatoses. Seborrheic verruca is also observed in patients with benign neoplasms, pregnancy, or human immunodeficiency virus infections.

Several treatments are available for eradicating verruca lesions including surgery, cryotherapy, electrocauterization, laser, or topical agents; however, the treatment strategy varies depending on disease location and severity, as well as the patient's immune status [3]. These are silent and slow growing lesions in healthy individuals, but can result in serious and rapid growth in immunosuppressed patients. Here we present two cases of verruca vulgaris and seborrheic verruca observed in patients with malignancy.

## 2. Case Presentation

### 2.1. Case 1

**Figure 1:**
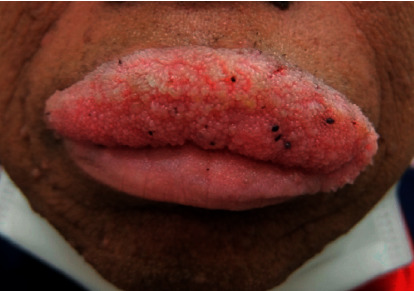
A rapidly growing papillomatous verruca originating on the lip and invading the palate.

A 61-year-old Japanese man visited our department for a verrucous formation on his lip that was growing rapidly and invading his palate (Figure 1). He also showed verrucous vegetation characterized by pigmentation, and a blackish brown mass with pruritus gradually developed on his axilla, posterior neck, and face. He had a history of hypertension, hyperuricemia, hyperlipidemia, gastrectomy and cholecystectomy for gastric cancer and was actively receiving chemotherapy for the treatment of multiple metastases. We diagnosed malignant acanthosis nigricans and verruca vulgaris. We intended to perform a skin biopsy of a patient with verrucous vulgaris to exclude verrucous carcinoma, squamous papilloma, condyloma acuminatum, and multifocal epithelial hyperplasia, but we could not obtain consent from the patient. The verruca was treated with cryotherapy without any response. Due to the serious phenotypes of his skin manifestations, we were anxious about the patient's immune status; however, his immune condition had been evaluated as “normal” by his physician on the basis of laboratory data including a complete blood count (CBC). Although his neutrophil function in phagocytosis and sterilization fell within the normal range, his natural killer (NK) cell activity was 0.3% (normal range: 8.9–29.5%). We issued a warning about his immunosuppressed status, but the patient died due to pneumonia and multiple organ failure.

### 2.2. Case 2

A 77-year-old man with a 37-year history of pustular psoriasis being treated with brodalumab, a human monoclonal antibody that binds to interleukin (IL) 17 receptor A (IL17R) with high affinity, was hospitalized for erythroderma and high fever. Chest computed tomography revealed pneumonia, a lung tumor, and mediastinal lymphadenopathy. Biopsy of the mediastinal lymph node with immunohistochemical study revealed lung adenocarcinoma. Furthermore, a head MRI revealed a brain metastasis. His performance status was 4, and he was not a candidate for chemotherapy.

**Figure 2:**
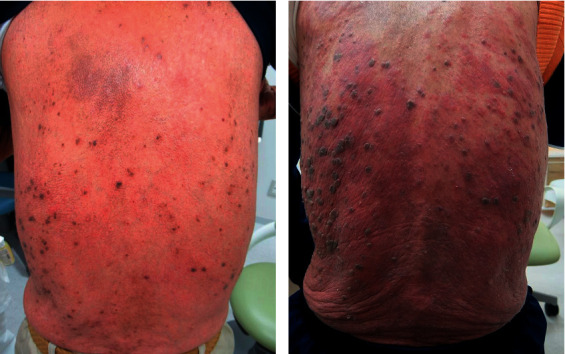
(a) Multiple flat black spots on the patient's back three months prior to admission. (b) Multiple seborrheic verruca on the patient's back on admission.

Multiple black-colored seborrhoic verruca appeared on his back, which rapidly increased in number and size within 3 months (Figures 2(a) and 2(b)). We speculated that these seborrhoic verruca were related to his lung cancer, a phenomenon known as the Leser-trélat sign. Although his CBC, including absolute neutrophil and lymphocyte counts, was within the normal range, his natural killer (NK) cell activity was 2.3% (normal range: 8.9–29.5%). We speculated the patient was in an immunosuppressed state. The patient's medical condition rapidly declined and he died within 2 months.

## 3. Discussion

NK cells are critical components of the innate immune system and do not express the T-cell receptor; they function in nonspecific cytotoxicity to virus-infected cells and cancer cells. NK cell receptors consist of activating and inhibitory receptors that determine susceptibility to virus infection. Inhibitory receptors recognize MHC-class I and, upon binding, will prevent cytotoxicity. Since virus-infected cells and cancer cells show down-regulated MHC-class I, NK cells attack target cells. One study found the percentages of NK cells were significantly decreased in patients with gastric cancer, and the percentages of tumor-infiltrating NK cells were positively correlated with survival and disease progression [4]. Brodalumab is an anti-IL-17RA antibody and hampers IL-17 signaling by blocking its receptor. IL-17 not only plays an oncogenic role in tumorigenesis by regulating tumor angiogenesis and enhancing tumor immune evasion but also exerts anti-tumor functions by enhancing NK cells and cytotoxic T lymphocyte (CTL) activation [5]. In the second case, brodalumab may have contributed to the measured reduction in NK cell activity.

Immune status is evaluated by checking CBC including white blood cells, but we often encounter viral infections such as cytomegalovirus in patients receiving anti-cancer drugs even with normal CBC. In these two cases, the CBC was within the normal range, but NK cell activity was low. By measuring NK cell activity, we noticed the fatal risk of lung infection in the first case, and we suspected seborrheic verruca related to lung cancer in the second case. Although CBC is normal, NK cell activity may be low in patients with abrogative skin rash, suggesting the importance of measuring NK cell activity.
